# In vivo evolution of drug-resistant *Mycobacterium tuberculosis* in patients during long-term treatment

**DOI:** 10.1186/s12864-018-5010-5

**Published:** 2018-08-29

**Authors:** Yuhui Xu, Fei Liu, Suting Chen, Jiannan Wu, Yongfei Hu, Baoli Zhu, Zhaogang Sun

**Affiliations:** 1 Institute of Chinese Materia medica, China Academy of Chinese Medical Science, Beijing, 100700 China; 2 National Tuberculosis Clinical Laboratory, Beijing Chest Hospital, Capital Medical University, Beijing, 101149 China; 3 CAS key Laboratory of Pathogenic Microbiology and Immunology, Institute of Microbiology, Chinese Academy of Science, Beijing, 100101 China; 4 Beijing Key Laboratory in Drug Resistant Tuberculosis Research, Beijing Tuberculosis & Thoracic Tumor Research Institute, Beijing, 101149 China

**Keywords:** Drug-resistant tuberculosis, Treatment, Genetic changes, SNP

## Abstract

**Background:**

In the current scenario, the drug-resistant tuberculosis is a significant challenge in the control of tuberculosis worldwide. In order to investigate the in vivo evolution of drug-resistant *M. tuberculosis*, the present study envisaged sequencing of the draft genomes of 18 serial isolates from four pre-extensively drug-resistant (pre-XDR) tuberculosis patients for continuous genetic alterations.

**Results:**

All of the isolates harbored single nucleotide polymorphisms (SNPs) ranging from 1303 to 1309 with *M. tuberculosis* H37Rv as the reference. SNPs ranged from 0 to 12 within patients. The evolution rates were higher than the reported SNPs of 0.5 in the four patients. All the isolates exhibited mutations at sites of known drug targets, while some contained mutations in uncertain drug targets including *folC, proZ,* and *pyrG*. The compensatory substitutions for rescuing these deleterious mutations during evolution were only found in RpoC I491T in one patient. Many loci with microheterogeneity showed transient mutations in different isolates. Ninety three SNPs exhibited significant association with refractory pre-XDR TB isolates.

**Conclusions:**

Our results showed evolutionary changes in the serial genetic characteristics of the pre-XDR TB patients due to accumulation of the fixed drug-resistant related mutations, and the transient mutations under continuous antibiotics pressure over several years.

**Electronic supplementary material:**

The online version of this article (10.1186/s12864-018-5010-5) contains supplementary material, which is available to authorized users.

## Background

Tuberculosis (TB) is a chronic infectious disease. In recent years, a significant drug resistance, especially multidrug resistance (MDR) and extensive drug resistance (XDR), has occurred [1]. In 2015, there were an estimated 480,000 new cases of MDR-TB and additional 100,000 persons with rifampicin-resistant TB. Moreover, 9.7% of MDR-TB was found to have XDR-TB, which further increases the difficulty of in treating TB [2]. The effect of genetic mutations in the drug resistance of *M. tuberculosis* has been extensively investigated in a large amount of isolates [3–7]; however, the genetic alterations during the course of the long-term treatment, especially at the late stage remain to be deciphered.

The genetic mutations in *M. tuberculosis* after the onset of disease have been analyzed, and the results vary based on the presence or absence of the anti-TB drug treatment. The selection process resulting from the antibiotic therapy may contribute to the occurrence of SNPs. Sun et al. reported that during the treatment, the drug susceptibility of the isolates changed from sensitive strains to resistant strains, and SNPs were acquired to confer the drug resistance [8], which was further confirmed in one XDR case evolved from a susceptible ancestor [9]. However, strains isolated from patients with recurrent tuberculosis exhibited few genetic mutations (0–6 SNPs) between the primary infection and relapse [10]. This was confirmed by the results of a retrospective observational study in which the genetic changes were rarely higher than five SNPs in 3 years and the estimated rate of change in DNA sequences was 0.5 SNPs per genome per year (95% CI 0.3–0.7) in longitudinal isolates [11].

In certain SNPs, the compensatory substitutions are common in drug-resistant TB, which can rescue the deleterious mutations during evolution, and to some extent reflect the transmissibility and prevalence of drug-resistant tuberculosis in a population. With an increase in the number of sequenced isolates, more and more compensatory mutation types have been identified in *M. tuberculosis* [3], especially the compensatory substitutions in RpoA and RpoC for rifampicin-resistant isolates. The compensatory substitutions are also found in the clinical isolates with different genotypes, such as Beijing genotype in northern of China [12], Russia [3, 13] and LAM4/F15/KZN in South Africa [14].

The previous reports have documented the evolution of drug-resistant *Mycobacterium tuberculosis* in serial *M. tuberculosis* isolates [8, 9, 15, 16]. Recently, Andrej et al. reported a view of the heterogeneity of MTBC populations in the host lung [17], but they only focused on the first 8 weeks of treatment and most of them were not MDR. The dynamics of bacterial populations within the host during the course of treatment is not well understood. In this study, deeper characterization of the microbial dynamics within the patient for several years was done based on our results, and a number of *M. tuberculosis* isolates were analyzed over time.

## Methods

### Sample selection from patients

Four patients were recruited from Beijing Chest Hospital. Patients were selected with refractory pulmonary tuberculosis that was confirmed by the clinical symptoms and pathophysiology analysis (based on the positive pathogen cultures and image of the lung by computed tomography). The hematology and blood chemistry, urine biochemistry, mixed bacterial infection, and drug allergic reaction status were observed throughout the routine clinical work.

Eighteen *tuberculosis* sputum samples from the four refractory tuberculosis patients were collected during treatment through routine clinical work and re-cultured in our laboratory for genome sequencing using Middlebrook 7H9 medium added with 10% oleic acid, albumin, dextrose, and catalase (OADC) (BD, MD, USA). Those bacteria were harvested, placed in 1 mL 15% glycerol, and saved in the specimen bank.

### Drug susceptibility tests

The drug susceptibility tests (DSTs) were performed using the absolute concentration method on Lowenstein-Jensen (L-J) slants. The concentration of each of the antibiotics, including isoniazid (INH), rifampin (RIF), streptomycin (SM), pyrazinamide (PZA), ethambutol (EMB), ofloxacin (OFX), capreomycin (CPM), p-aminosalicylic acid sodium (PAS), ethionamide (ETH), and amikacin (AmK) was indicated in the description [18].

### Molecular typing

The bacterial genotypes were determined using a commercial kit (Isogen Bioscience BV, Maarssen, The Netherlands) according to the reported method by Kamerbeek et al. [19]. The number of tandem repeats at each locus in the isolates was determined based on the number of whole repeats in the PCR product of the size estimated from the gel. PCR for the 12 chosen loci was repeated and compared within and between gels to ensure consistent estimation of the size and tandem repeat copy number [20].

### Genome sequencing and assembly

The genomic DNA of the bacteria was extracted according to the manufacturer’s guidelines (Qiagen, Beijing, China) [18]. A 500-bp paired-end library was constructed for each purified DNA sample according to the standard Illumina paired-end protocol with a low-cycle polymerase chain reaction during fragment enrichment. The sequencing was performed on the Illumina Genome Analyzer platform for either 100 or 150 cycles. The low quality reads were filtered using the DynamicTrim and LengthSort Perl scripts in SolexaQA [21]. The short reads were assembled with SOAPdenovo [22] with various length of kmer, a genome assembler developed specifically for next-generation short-read sequences, and the gaps were filled with GapCloser from SOAP after assembling.

The drug-resistant strain *M. tuberculosis* 11,495 was sequenced using PacBio Single-molecule real-time (SMRT) sequence technology. We used the Hierarchical Genome Assembly Process (HGAP.3) algorithm in SMRT Portal (version 2.2.0) to perform the genome assembly. After sequencing and quality-filtering, 330,992 reads were obtained with a mean length of 3905 bp totaling 1,292,425,796 bp. The complete genome sequence of *M. tuberculosis* 11,495 has a length of 4,428,395 bp and a G + C content of 65.6%.

### Mutation detection

*M. tuberculosis* H37Rv is the most studied strain of tuberculosis in research laboratories. However, it is becoming apparent that use of H37Rv as a sole reference genome in analyzing clinical isolates presents some limitations in investigating *M. tuberculosis* [23]. Hence, we included the genome sequence of a Beijing lineage 11,495 genetically close to our strains in our analyses, in addition to H37Rv. First, the short reads were aligned with two reference genomes using the SOAP2 program [24], which included the standard reference strain *M. tuberculosis* H37Rv (GenBank AL123456), and the other strain 11,495. Then, SOAPsnp was used to score the SNPs from the aligned reads [25]. The SOAPsnp results were filtered as follows: (1) the read coverage of the SNP site was greater than three, (2) the Illumina quality score of each allele was greater than 30, and (3) the number of mapped best bases was more than two times the number of all mapped second-best bases. SNPs located in PE/PPE and PE-PGRS gene families that might cause incorrect read alignment were also excluded. In addition, SNPs showing sequencing and analysis noise patterns were manually removed. For deletion analysis, we used the results from the aligned reads, and the regions covered by less than three reads were considered as the deletion regions. To confirm SNPs and deletion analysis, we also performed a genome comparison analysis with results from genome assembly by Mauve [26] using default parameters. The correlation coefficient between the number of SNPs and the duration of the treatment was analyzed by the Pearson method. A *p* value of < 0.05 was considered statistically significant. Fisher’s exact tests [27] were used to assess the statistical significance of the difference in SNPs between our sequenced genomes and the drug-resistant reference Beijing genomes. COG annotation was performed using the BLAST software against the COG database. COG enrichment analysis was determined using Fisher’s exact test by comparing the prevalence of a target group of genes assigned to a specific COG category to the prevalence of genes in the whole genome.

### Phylogenetic analysis

Maximum likelihood phylogenetic tree of the *M. tuberculosis* isolates was created using the SNPs of the whole genome sequences by kSNP3 with a k-mer length of 19 [28]. A median joining network can be used to infer intraspecific phylogenies where small genetic mutations are expected. It resolves all possible evolutionary paths connecting the considered taxa and postulates new nodes. Hence, a median-joining tree was created using the Population Analysis with Reticulate Trees (PopArt v1.7) [29].

## Results

### Characteristics of the strains

Four refractory tuberculosis patients without adjunctive surgery were included in this study. Only one patient was experiencing a first-time hospitalization; the others were re-treatment patients with a long history of tuberculosis before being hospitalized (Table 1). All of the patients exhibited more than 10% of weight loss during their hospital stay. The abnormal blood chemistry indices included alpha-hydroxybutyric acid and C-reactive protein. In addition, all patients exhibited abnormal hematology indices with higher erythrocyte sedimentation rate and fibrinogen concentration. All patients had normal urine biochemistry.

**Table 1:** Patient clinical data

NO.	*Diagnosis*	*DH(y)*	*We*i*ght loss(*kg)	HE	Blood Chemistry	ESR	FC	ID	UB	ARD	OBI	Survival
A	IPT	45	9/64 (14.06%)	N	LAHTA, HCRP	11.37 ± 6.23	4.45 ± 1.23	No	N	RFP, PZA	AHSC, NC	YES
B	CFPT	12	8/58 (13.79%)	N	NAHBA, HCRP	21.37 ± 8.23	6.45 ± 2.23	No	N	NO	AHSC, NC	YES
C	IPT	28	17/75 (22.67%)	N	LAHBA, HCRP	35.38 ± 12	6.06 ± 1.60	No	N	NO	AHSC, NC	YES
D	IPT	0	14/58 (24.13%)	N	NAHBA, HCRP	26.67 ± 2.52	5.64 ± 1.12	No	N	NO	AHSC, NC	YES

*DH(y)* disease history (years), *HE* hematology, *ESR* erythrocyte sedimentation rate, *FC* fibrinogen concentration, *ID* immunodeficiency, *UB* urine biochemistry, *ARD* allergy reaction drugs, *OBI* other bacterial infection, *IPT* infiltrative pulmonary tuberculosis, *CFPT* chronic fibrocavenous pulmonary tuberculosis, *LAHBA* low alpha-hydroxybutyric acid, *NAHBA* normal alpha-hydroxybutyric acid, *HCRP* high C- reactive protein, *AHSC* α-hemolytic streptococcus, *NC* Neisser’s coccus, *N* normal

The implemented treatment regimens followed the WHO guidelines according to the best drug combinations and dosing schedules for multidrug resistance tuberculosis (MDR-TB) and extensive drug resistance tuberculosis (XDR-TB) [30, 31]. However, the patients were still not cured in an adequate amount of time and three of the four cases were resistant to the fourth and fifth group of the anti-TB drugs, such as ETH and PAS (Table 2**)**. Moreover, the drug resistance patterns of the four patients increased, with the progression in treatment. Drug susceptibility tests showed that the initial strains from four patients were pre-XDR before treatment but later became XDR by the end of the study.

**Table 2:** Medication and drug resistance of the phenotypes and genotypes of four refractory tuberculosis patients

		Patient A^#^					Patient B			Patient C					Patient D				
Drug	gene^a^	A1^#^	A2	A3	A4	A5	B1	B2	B3	C1	C2	C3	C4	C5	D1	D2	D3	D4	D5
H	katGa	T326 M	T326 M	T326 M	T326 M	T326 M	Y98S	Y98S	Y98S	S315 T	S315 T	S315 T	S315 T	S315 T	S315 T	S315 T	S315 T	S315 T	S315 T
	inhA promotor	−15, C-T	−15, C-T	− 15,C-T	−15, C-T	− 15,C-T				−15, C-T	−15, C-T	− 15, C-T	−15, C-T	− 15, C-T					
R	rpoB	H526L	H526L	H526L	H526L	H526L	H526D	H526D	H526D	D516V	D516V	N373D/ D516V	N373D/ D516V	N373D/ D516V	S531 L	S531 L	S531 L	S531 L	S531 L
Z	pncA	A87T	A87T	A87T	A87T	A87T	A87T	A87T	A87T	A87T	A87T	A87T	A87T	A87T	A87T	A87T/ L120P	A87T/ L120P	A87T/ L120P	A87T/ L120P
E	embB	M306 V	M306 V	M306 V	M306 V	M306 V	Y319S	Y319S	Y319S	G406A	G406A	G406A	G406A	G406A	M306 V	M306 V	M306 V	M306 V	M306 V
	Rv3806c							A249G	A249G						F140 V	F140 V	F140 V	F140 V	F140 V
	Rv3756c (*proZ)*	G56D	G56D	G56D	G56D	G56D													
	Rv1699 (*pyrG)*				G407D	G407D													
S	rpsL	K88R	K88R	K88R	K88R	K88R										K43R	K43R	K43R	K43R
	rrs						c1402t/ a1482g	c1402t/ a1482g	c1402t/ a1482g	a514c c1402t	a514c c1402t	a514c c1402t	a514c c1402t	a514c c1402t	a1401g	a1401g	a1401g	a1401g	a1401g
LFX	gyrA	D94G	D94G	D94G	D94G	D94G	A90V	A90V	A90V	A90V	A90V/ S91P	A90V/ S91P	A90V/ S91P	A90V/ S91P		D94N	D94N/ A90V	D94N/ A90V	D94N/ A90V
PAS	Rv2447c (folC)		I43T	I43T	I43T	I43T	E153G	E153G	E153G										
Amk	Rv2416c (eis)				P2L	P2L													
MDR+	Rv1129c	P131T	P131T	P131T	P131T	P131T	P131T	P131T	P131T	D160A	D160A	D160A	D160A	D160A	P131T	P131T	P131T	P131T	P131T
CPM/Amk	Rv0323c		V215 L	V215 L	V215 L	V215 L	V215 L	V215 L	V215 L	V215 L	V215 L	V215 L	V215 L	V215 L	V215 L	V215 L	V215 L	V215 L	V215 L
LFX/ETH	Rv0404 (*fadD30)*							L10R	L10R	L10R	L10R	L10R	L10R	L10R	L10R	L10R	L10R	L10R	L10R
CPM/Amk/ LFX/ETH	Rv0565c						F68I	F68I	F68I							T402 M	T402 M	T402 M	T402 M
ETH + Amk	Rv2080 (*lppJ*)						S82^a^	S82^a^	S82^a^	S82^a^	S82^a^	S82^a^	S82^a^	S82^a^					
ETH + Amk + CMP + S	Rv2447c (*folC*)		I43T	I43T	I43T	I43T	E153G	E153G	E153G										
Drugs ever used		H R Z E	E Pa LFX PAS Amk	E Clr Pa LFX PAS Amk	E Clr Pa LFX PAS Amk	E Clr Pa LFX PAS Amk	R Z CPM LFX ETH Clr	R Z CPM LFX ETH Clr	E Clr PAS MFX Rfb	E Amk PAS ETH LFX	E Amk PAS ETH LFX	E Amk PAS ETH LFX	E Amk PZA PAS ETH LFX	E Amk PZA PAS ETH LFX	E Z Amk Clr CPM LFX	E Z Amk Clr CPM LFX	E Z S Amk Clr CFZ MFX	E Z S Amk Clr CFZ MFX	E Z S Amk Clr CFZ MFX
Resistant to		H R S LFX ETH	H R S LFX ETH PAS	H R S LFX ETH PAS	H R S LFX Amk ETH PAS	H R S LFX Amk ETH PAS	H R LFX ETH PAS	H R LFX ETH PAS CPM	H R E S LFX ETH PAS CPM	H R CPM	H R Lfx CPM	H R Lfx CPM	H R Lfx CPM	H R S Lfx CPM	H R LFX ETH PAS	H R LFX Amk ETH PAS	H R LFX Amk ETH PAS	H R S LFX Amk ETH PAS	H R S E Amk LFX ETH PAS CPM

*H* isoniazid, *R* rifampin, *S* streptomycin, *Z* pyrazinamide, *E* ethambutol, *LFX* levofloxacin, *MFX* moxifloxacin, *PAS* p-aminosalicylic acid sodium, *Amk* amikacin, *ETH* ethionamide, *CPM* capreomycin, *Clr* clarithromycin, *CFZ* Clofazimine

^a^drug-resistant related genes mainly based on the report by Zhang et al. [5]

^#^A, B, C, and D indicate the four cases, and the number following them indicates the different isolates

### Molecular genotype and whole genome sequencing

PCR-based variable-number tandem repeat (VNTR) analysis was performed using the 12-MIRU-locus method and two patterns were identified, 1241 2728 3422 for isolates from patient A and 2261 2631 3321 for isolates from patients B, C and D. Both the Spoligotyping (octal codes: 000000000003771) and genome sequencing results showed that all the isolates belonged to the Beijing lineage.

The basic whole-genome sequencing statistics are shown in Additional file 1: Table S1. For each sample, 2.0 to 5.8 million 150-bp or 100-bp paired-end reads were obtained, which corresponded to an average sequencing depth ranging from 138 to 270-fold. The GC content of the genomes was approximately 65.5%, as expected for the species. The size of the genomes varied from 4.26 to 4.33 Mb.

### Phylogenetic analysis of *M. tuberculosis* isolates

**Fig. 1:**
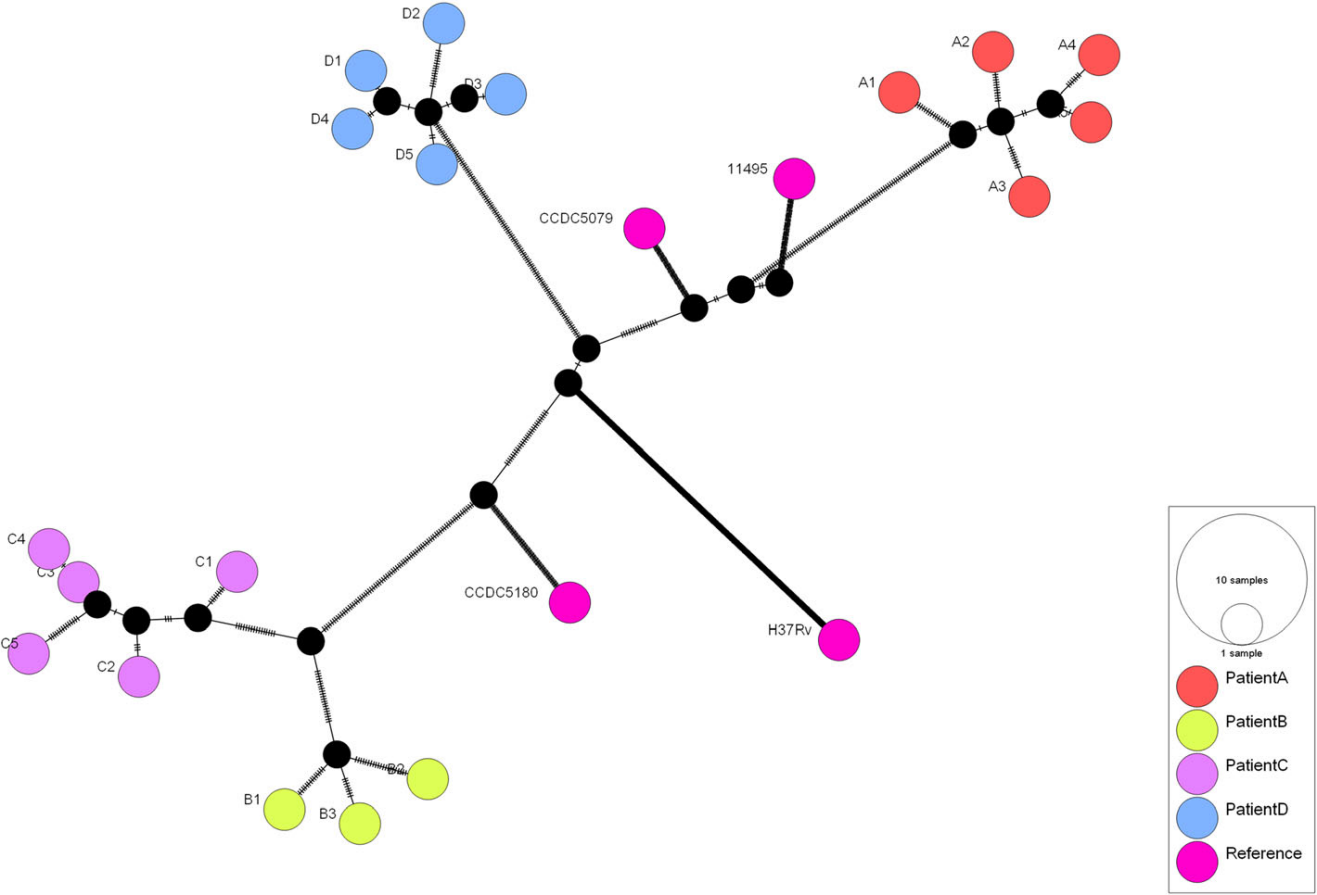
Median-joining networks based on SNPs in *Mycobacterium tuberculosis* isolates showing microevolution events. The nodes of the networks correspond to the different MTB isolates. The color inside each circle represents the host. Each short line along the lines, linking the nodes corresponds to a single-nucleotide polymorphism (SNP) detected between the variants in the connected nodes

A median-joining tree was created based on the SNPs from draft genome sequences of the 18 clinical *M. tuberculosis* isolates and the complete genomes of four additional *M. tuberbulosis* strains (Fig. 1). All clinical isolates from the same patients formed separate clades, which indicated that the successive isolates were derived from the same ancestor strain.

#### SNP and Indel detection

**Fig. 2:**
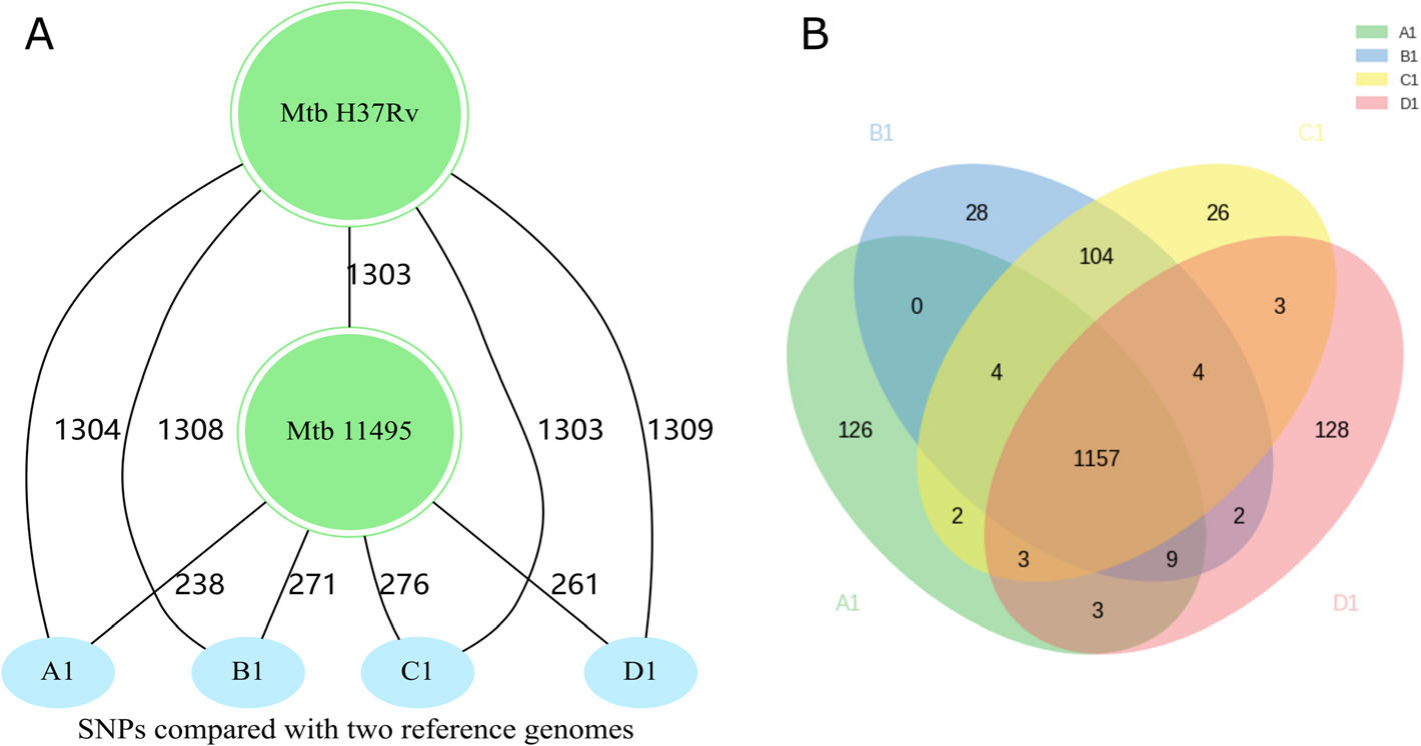
SNPs of the bacterial isolates collected for each patient. **a** The number of SNPs was assessed relative to H37Rv (NC_000962.3) and 11,495. The number on the line indicates the number of SNPs between the two isolates. **b** Venn diagram of different SNPs in each patient relative to reference H37Rv

With *M. tuberculosis* H37Rv (ATCC 27294) as the reference, the sequencing coverage was approximately 99%, and all of the isolates harbored SNPs ranging from 1303 to 1309 (Fig. 2a**,** Additional file 1: Table S1). TC, AG, GA, and CT transitions were found to be the most frequent SNP types (Additional file 2: Figure S1), and the mean transversion/transition ratio was 0.60. With *M. tuberculosis* 11,495 as the reference, the four isolates harbored SNPs ranging from 238 to 276 (Fig. 2a).

Venn diagram showing the SNPs distribution in four isolates under study compared with the H37Rv demonstrates that all the strains used in the experimentation appeared to be quite similar (Fig. 2b): 1157 SNPs were common, while each isolate possessed a few individual SNPs.

**Fig. 3:**
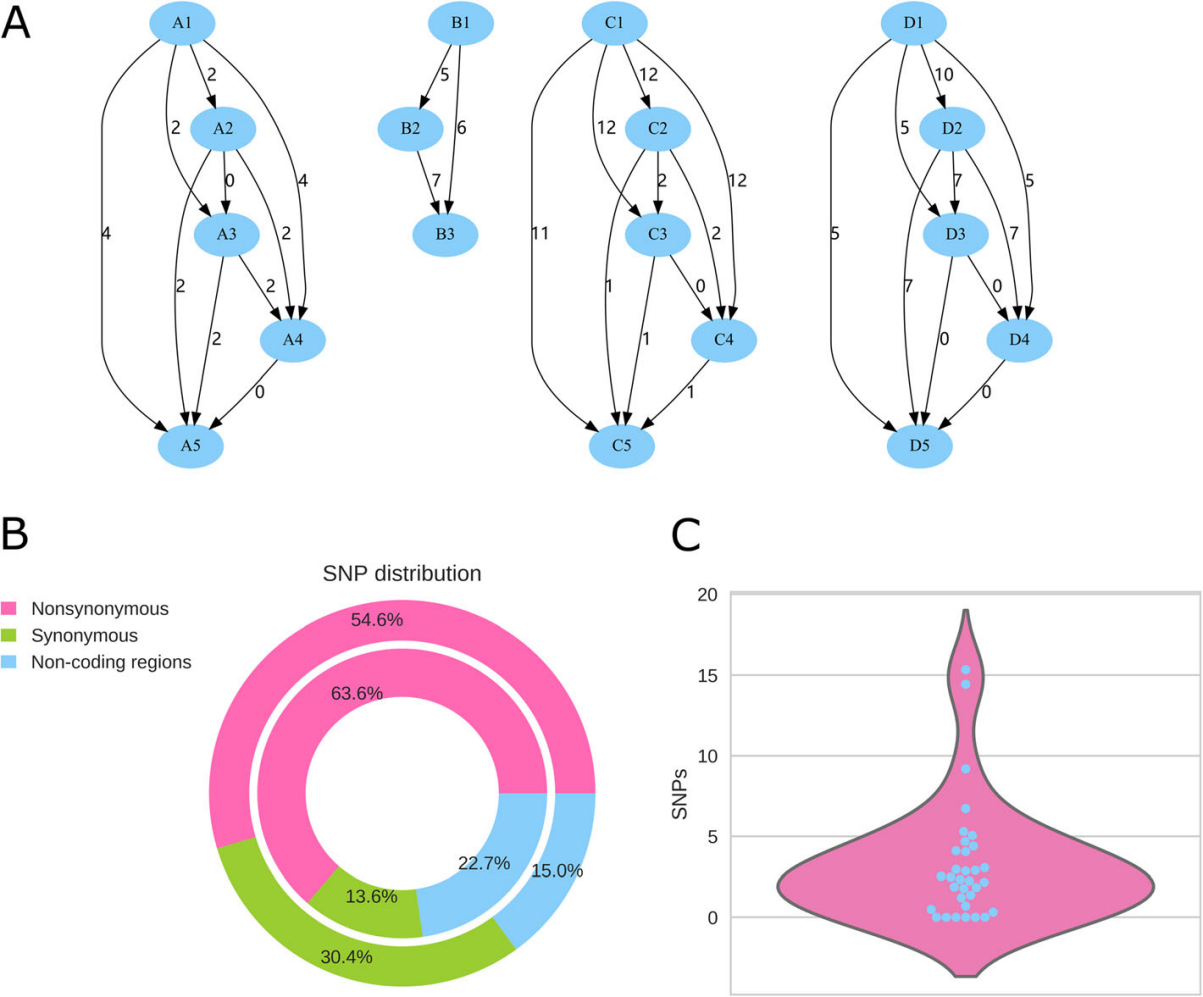
Intra-patient evolution and mutation rates. **a** The numbers of SNPs of the bacterial isolates at different stages of treatment for each patient. A, B, C, and D represent the four selected patients. The number on the short line indicates the numbers SNPs between two isolates at different time points of treatment of the same patient. **b** Pie chart depicting distribution of SNPs. Outer: patients compared with H37Rv; Inner: intra-patient. **c** Violin plot of calculated pairwise mutation rates per year between any pair of strains

The intra-patient isolates exhibited a low SNPs variation ranging from 0 to 12 (Fig. 3a). There were 63.6% nonsynonymous, 13.6% synonymous, and 22.7% noncoding SNPs in intra-patient SNPs; while there were 54.6% nonsynonymous, 30.4% synonymous, and 15.0% noncoding SNPs when compared with H37Rv (Fig. 3b). Mutation rate is the number of SNPs between any two paired isolates per the calendar year, was calculated for each patient. The mean mutation rate was 3.2 SNP per genome per year (Fig. 3c). There was no statistically significant relationship between the treatment time and the SNP number (*p* < 0.05).

**Fig. 4:**
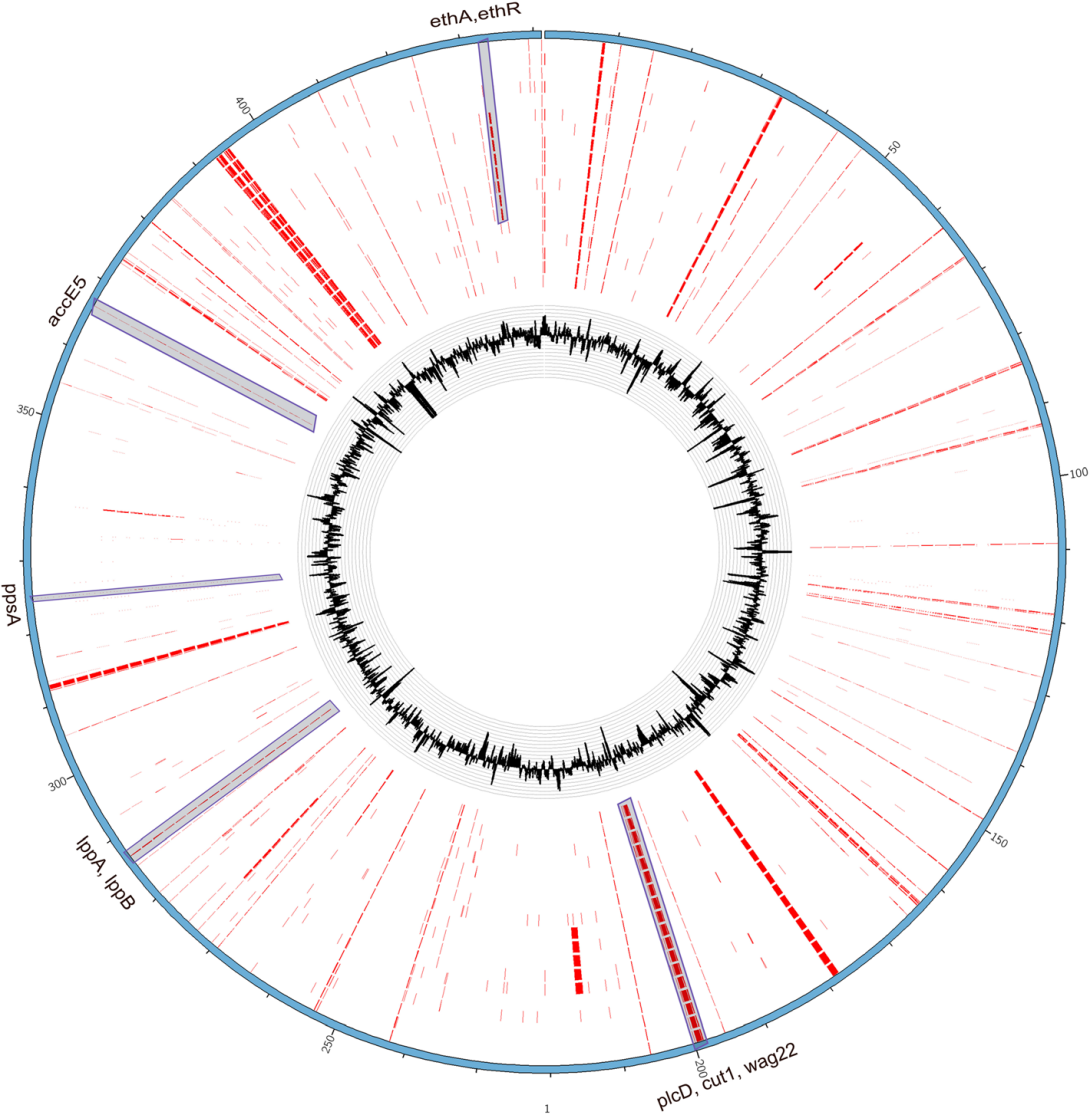
Comparison of the missed regions among the sequenced genomes. A circular map of the regions (Red color) missed when mapping the raw reads of the sequenced strains against the H37Rv genome (NC_000962.3). Inside track within the map displays a plot of G + C contents. In outer strain circles, from the inside out, include the following missed regions in those isolates of A1, A2, A3, A4, A5, B1, B2, B3, C1, C2, C3, C4, C5, D1, D3, D4, and D5. The circular map was constructed using Circos, which is a software package for visualizing data and information (http://circos.ca/). Genes in the main missed regions were showed (Table 3), not including those belonging to PE or PPE family that contain large, near perfect repeats that create a high likelihood of sequencing and assembly error

When compared to H37Rv, some deleted genes were also identified in more than one of the tested patients (Fig. 4). *cut1*, *plcD*, and *wag22* genes in all of the isolates were found to be completely deleted.

### Mutations related to drug resistance

In this study, the nonsynonymous mutations were found in many drug target genes (Table 2**).** The nonsynonymous mutations related to current anti-TB drugs in *katG*, *pncA*, *gyrA*, *rpsl*, *rpoB*, *embB*, *ethA, rrs,* and *Rv3806c* were already confirmed [5, 11]. The nonsynonymous mutations in *katG*, *rpoB*, *pncA*, *embB,* and *gyrA* were found in all of the 18 isolates, and drug-resistance related mutations in *rpsl*, *rrs*, *ethA, Rv3806c* and *inhA* promoter were found in the isolates from certain patients. No mutation was found in *rpsA* and *panD* for PZA resistance, in *ribD* and *thyA* for PAS resistance, in *gidB* for SM or Amk resistance, in *embA*, *embC*, *embR*, *iniAC* for EMB resistance, and in *ahpC*, *fabG1* and *inhA* for INH resistance (Table 2). Some nonsynonymous mutations that were inferred for drug resistance [32] were also found in part among the isolates, such as *Rv2447c (folC), Rv3756c (proZ), Rv1699 (pyrG), Rv1129c, Rv0323c, Rv0404 (fadD30), Rv0565c, Rv2080 (lppJ)* and *Rv3862c (whiB6)*. We analyzed the occurrence of compensatory substitutions in RpoA and RpoC in all the sequenced 18 isolates and found that the compensatory substitutions exist only in RpoC at I491T, in isolates from patient D.

### Transient mutation

Apart from the fixed mutations that were related to the drug resistance, several transient mutations was also found in all of the patients (Additional file 3: Table S2). A micro-heterogeneity at some related genome sites of the isolates was found on further examination of the transient mutations based on the raw reads. Interestingly, the same base positions that determined this microheterogeneity were also found in the isolates from different patients.

Next, we investigated all the nonsynonymous SNPs to identify the mutations that could be involved in infection and persistence in all patients. The isolates in patient A harbored a P2L mutation in *eis* (Rv2416c) which shows to enhance the intracellular survival of *M. tuberculosis* [33, 34]. In patient C, we found a C451Y mutation in *ponA2* (Rv3682), which is involved in the adaptation of *M. tuberculosis* to dormancy [35].

### Confirmation of the SNPs and Indels with published data

**Fig. 5:**
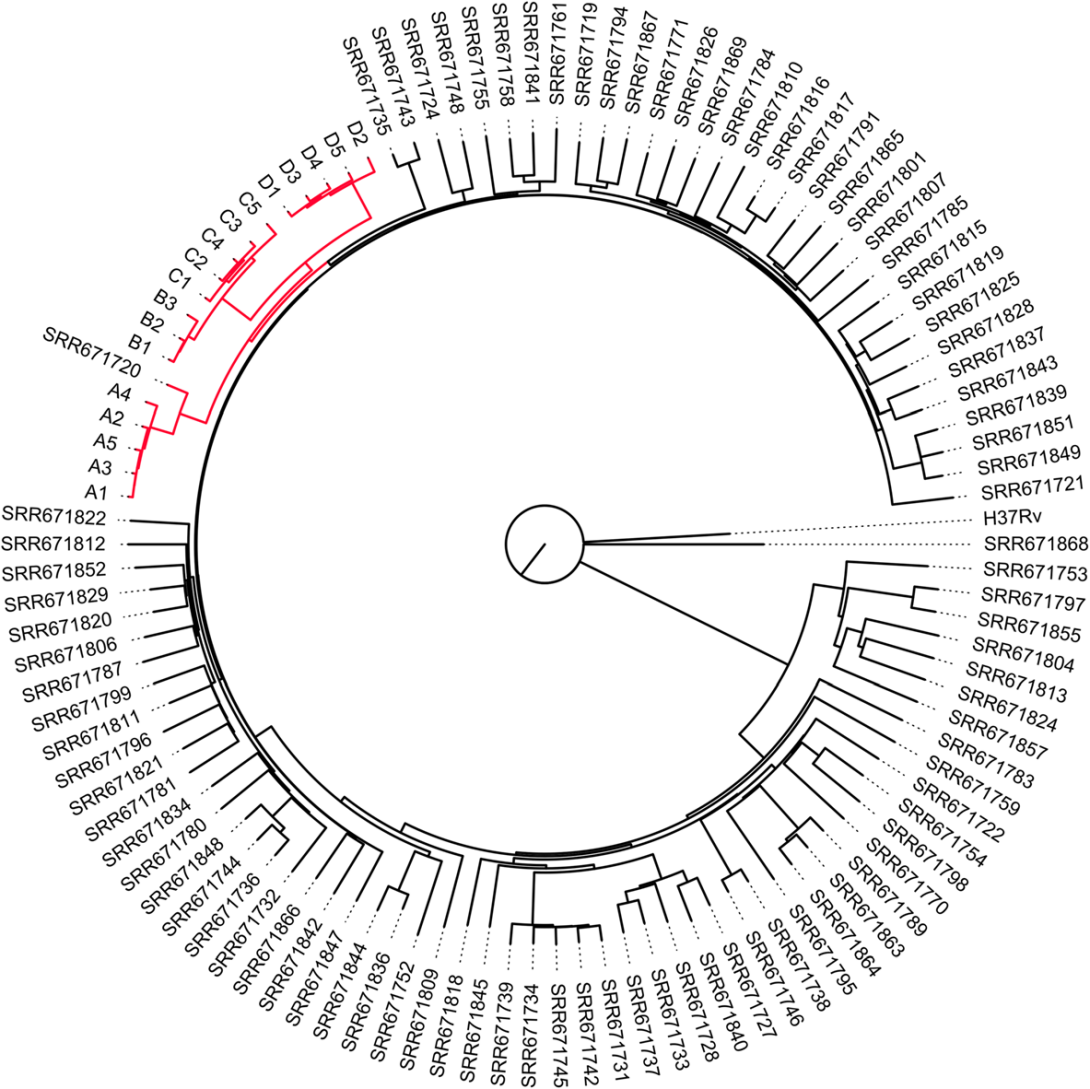
Microevolution of the tested refractory TB population. Maximum likelihood phylogenetic trees of the *M. tuberculosis* drug-resistant isolates were created using the SNPs of the whole genome sequences by kSNP3 with a k-mer length of 19. The red branch includes all of our sequenced refractory TB strains

We downloaded all the raw read sequences reported in China [5] from NCBI’s Sequence Read Archive (SRA) by SRA Toolkit and performed de novo genome assembly for all samples by SOAPdenovo [22] with various length of kmer. In order to find the specific SNPs of the tested isolates with Beijing genotypes in China, their draft genome sequences were compared to the reference drug-resistant strains. The phylogenetic trees demonstrated that the tested 18 bacterial isolates are evolutionarily close (Fig. 5). Ninety three SNPs exhibited significantly different percentages (adjusted *p*-value< 0.01) between our sequenced genomes and the reference drug-resistant Beijing genomes (Additional file 4: Table S3). Interestingly, we found two nonsense mutations, 1 coding sequence substitution c. 397G > T mutation occurred in Rv0768 (*aldA*) and one c. 245C > A mutation occurred in Rv2080 (*lppJ*).

We further analyzed the distribution of SNPs according to the different categories of the Clusters of Orthologous Groups (COG). Secondary metabolites biosynthesis, transport, and catabolism (class Q), Lipid transport and metabolism (class I), and Energy production and conversion (class C) were the most frequent COG categories (Additional file 5: Figure S2). In addition, we found that they were significantly enriched in Secondary metabolites biosynthesis, transport, and catabolism (class Q; *P* value = 0.048).

In order to confirm the deletions, these results were compared to the reported genome sequences in China [5]. Almost all of the three genes (*cut1*, *plcD*, and *wag22*) were partially deleted in all of the Beijing genotype isolates, and in few of the non-Beijing genotype isolates. There was no difference in the frequency of deletion of genes among the drug-resistant and drug-sensitive isolates. The deletions appeared to be the marker of Beijing genotype rather than being the cause of a long-term TB history.

## Discussion

Drug-resistant tuberculosis is a serious threat to public health worldwide. The varied genetic mutations in *M. tuberculosis* after its onset depend on the microenvironment in which the tuberculosis bacilli exist. The selection process resulting from the antibiotic therapy may be one of the main reasons for the occurrence of SNPs. The occurrence of SNPs in the genomes from the patients in whom the drug susceptibility changed from sensitive to resistant strains is well known [8, 9]. Recent studies involving re-sequencing of a large number of clinical isolates have also provided important insights into the complex evolution patterns of the TB pathogen [3, 4, 36, 37]. This study further confirmed the genetic changes when the isolates in pre-XDR patients were treated with more complex regimens in an attempt to explain the prolonged histories of some tuberculosis patients (Table 2**)**.

The genomic changes of the isolates within the individuals were significantly different. Only a few mutations, were found between baseline and re-infection, and there were no different sites between the baseline and relapse strains in 82% relapse cases [10]. In longitudinal isolates from 30 individuals and 25 families, the estimated rate of change in DNA sequences was 0.5 SNPs, per genome, per year (95% Confidence interval 0.3–0.7), with the divergence rarely higher than five SNPs in 3 years [11]. However, Ford et al. suggested that the rate of change in DNA sequences is a constant of 0.5 SNP per genome per year in latent, active, or re-activated disease over the same period of time [38]. In this study, the mean mutation rate was 3.2 SNP per genome per year (Fig. 3c). This indicates that the complex treatments might increase the genetic changes including the compensatory substitutions and the non-coding regions (Fig. 3b). Furthermore, based on the previous reports, we found that the SNPs of each isolate ranged from 1300 to 1500 bases [10, 39], while the SNPs among the strains with Beijing genotypes range from 200 to 500 bases [5, 19]. The SNPs of the intra-isolates were about 22.4 bases at the early sensitive stage [10].

Transient mutations were reported early in a case that evolved from a susceptible ancestor to XDR-TB [9]. The genetic loci, in this case, were not stable and changed their priority of bases under the antibiotic circumstance [9], implying the microheterogeneity in the cultured sputum population. In this study, many loci with microheterogeneity were found in the genome of the isolates at each time point and were repeatedly found in the isolates from different patients. The transient mutations were observed higher in the nonsynonymous (63.6%) and non-coding regions (22.7%) in intra-patient than that compared with H37Rv, with 54.6% nonsynonymous and 15.0% non-coding SNPs **(**Fig. 3b**)**. The transient mutations suggest that the unstable genetic sites exist in the *M. tuberculosis* genome with the probability of a strong positive selection under the pressure of antibiotics.

The compensatory substitutions are common in drug-resistant TB that reflects to some extent the transmissibility and prevalence of drug-resistant tuberculosis in a population. With an increase in the number of sequenced isolates, more and more compensatory mutation types were observed in *M. tuberculosis* [3, 13, 40, 41]. For rifampicin-resistant isolates, the occurrence of compensatory substitutions in RpoA and RpoC were frequently found in isolates carrying the *rpoB* S450 L mutation (equivalent to *Escherichia coli* S531 L) [3, 13, 40, 42]. In this study, compensatory substitutions were only found in RpoC (I491T) in the isolates from patient D.

A large number of mutations related to the drug resistance have been found worldwide. In this study, the isolates from four pre-XDR patients had mutations in genes like *katG* and *inhA* promoter for INH, *rpoB* for RIF, *rrs* and *eis* for Amk/CPM, and *gyrA* for LFX [5, 37]**.** Some other mutations related to the resistance of EMB (*embB*, *Rv3806c*, *proZ*, *pyrG*), PZA (*pncA*), and PAS (*folC*) were also found in some of the isolates, with the genotype in keeping with the phenotype (Table 2). In addition, some genes related to the multidrug resistance were listed in Table 2, such as Rv3756c (*proZ*), Rv1699 (*pyrG*), Rv1129c, Rv0323c, Rv0404 (*fadD30*), Rv0565c and Rv2080 (*lppJ*), which need to be further verified by molecular and biochemical methods.

In this study, we tried to find the characteristics of the SNPs and Indels specific to the four refractory pre-XDR TB patients, in comparison with the not refractory pre-XDR TB published data [5]. The phylogenetic tree demonstrated that the isolates in this study are phylogenetically close to each other (Fig. 5), indicating that the SNPs found in the refractory 18 tuberculosis isolates were potentially specific, of which 93 SNPs exhibited significantly different percentages (Additional file 4: Table S3). Although the main deletions in *cut1* [43], *plcD*, and *wag22* occurred in all of the *M. tuberculosis* populations*,* our data showed negative results, indicating that these deletions were specific for drug-resistant isolates, and they might be characteristics of the Beijing lineage (Table 3).

**Table 3:** Comparison of the deleted genes that occurred in the Beijing genotype and the non-Beijing genotype isolates

Gene name^a^	Gene length (bp)	Beijing genotype (n, %)		Non- Beijing Genotype (n, %)	
		Resistant (*n* = 91, %)	Sensitive (*n* = 21)	Resistant (*n* = 26)	Sensitive (*n* = 23)
*cut1*	525	91, 100	20, 95.24	13, 50.00	14, 60.87
*plcD*	843	90, 98.91	20, 95.24	4, 15.38	7, 30.43
*wag22*	2745	78, 85.71	21, 100	21, 80.77	16, 69.56

#The genome sequence of Mycobacterium tuberculosis H37Rv (NC_000962.3) was used as reference

^a^Deletion occurred in all of the populations in all three genes

## Conclusions

In summary, four pre-XDR tuberculosis cases were enrolled in this study to test the whole genome changes of the 18 serial isolates from these patients during the elaborate treatment. The results indicate that each patient with a long history of TB is not infected by mixed strains (pathogen population). The SNPs found by whole genome sequencing showed transient and fixed mutations. Mutations related to the drug-resistance were fixed under the continuous drug pressure. We also found a transient mutation in some cases, suggesting that the treatment regimen could eradicate some bacterial population at different stages, except for the mutations related to the drug resistance. Of all the SNPs and Indels, 93 SNPs exhibited significantly different percentages in this study. These results indicate that the genetic changes might be partly responsible for refractory pre-XDR TB in the four selected TB cases.

## Additional files

Additional file 1: **Table S1.** Sequencing statistics of the 18 *M. tuberculosis* isolates. Note: A, B, C, and D indicate the four cases, and the number following them indicates the different isolates. Sampling Time means the time that the samples collected from the four cases. H37Rv coverage (%) means the percentage of the H37Rv genome covered by mapping reads. *The number of SNPs was assessed relative to H37Rv (NC_000962.3). (XLSX 11 kb)

Additional file 2: **Figure S1.** Boxplot graph showing the different types of SNP mutations. (PNG 117 kb)

Additional file 3: **Table S2.** Detailed information of SNPs within four patients. (XLSX 14 kb)

Additional file 4: **Table S3.** Ninety three SNPs exhibited significantly different percentages (adjusted *p*-value< 0.01) between our sequenced genomes and the reference drug-resistant Beijing genomes. (XLSX 32 kb)

Additional file 5: **Figure S2.** Distribution of SNPs according to the Clusters of Orthologous Groups (COG) classification. (PNG 41 kb)

## Abbreviations

Amk: Amikacin
COG: Clusters of Orthologous Groups
CPM: Capreomycin
DSTs: Drug susceptibility tests
EMB: Ethambutol
ETH: Ethionamide
INH: Isoniazid
LFX: Levofloxacin
MDR: Multidrug resistance
OFX: Ofloxacin
PAS: p-aminosalicylic acid sodium
PZA: Pyrazinamide
RIF: Rifampin
SM: Streptomycin
SNPs: Single nucleotide polymorphisms
TB: Tuberculosis
VNTR: Variable-number tandem repeat
XDR: Extensive drug resistance
