# Supplementary figures and images for: In vivo evolution of drug-resistant Mycobacterium tuberculosis in patients during long-term treatment
Source: BMC Genomics. 2018 Aug 29;19:640. doi: 10.1186/s12864-018-5010-5 (PMC6116439; doi:10.1186/s12864-018-5010-5)

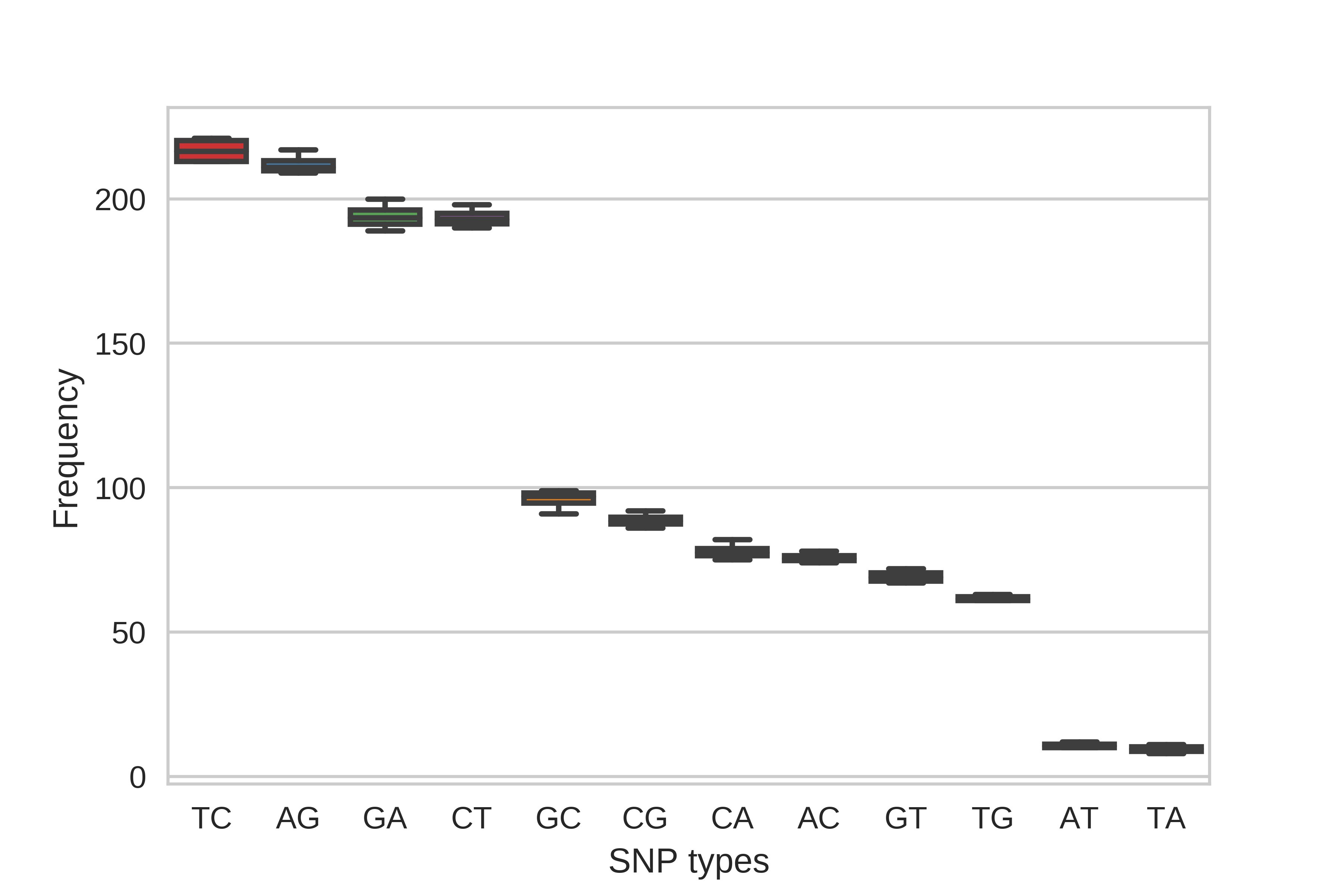

Supplement: Supplementary file 2 — Figure S1. Boxplot graph showing the different types of SNP mutations. (PNG 117 kb) [file 12864_2018_5010_MOESM2_ESM.png]

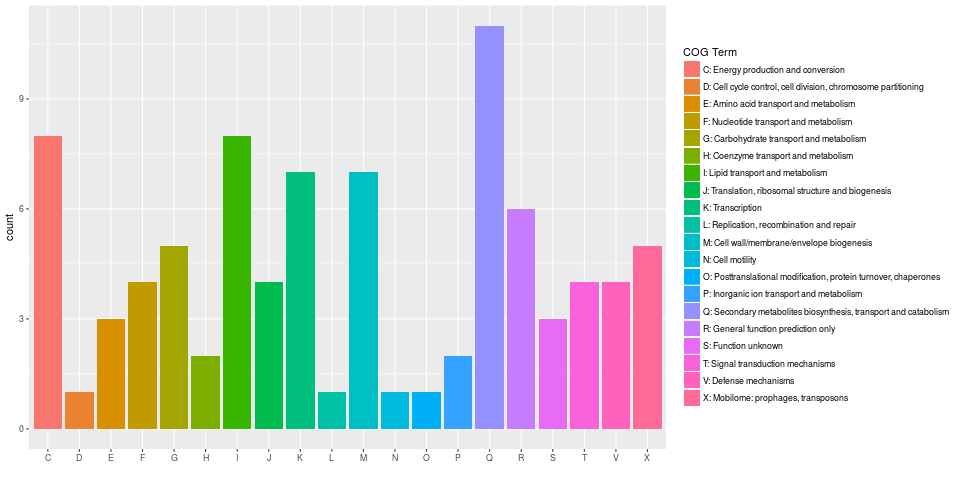

Supplement: Supplementary file 5 — Figure S2. Distribution of SNPs according to the Clusters of Orthologous Groups (COG) classification. (PNG 41 kb) [file 12864_2018_5010_MOESM5_ESM.png]
